# Epidemiological Shifts in Visceral Leishmaniasis Incidence, Relapse, and Mortality in Brazil, 2007–2023: Analysis Using the National Notifiable Diseases Information System

**DOI:** 10.1093/ofid/ofag035

**Published:** 2026-02-17

**Authors:** Dawit Getachew Assefa, Glaucia Cota, James P Wilson, Jessica Andretta Mendes, Caitlin Naylor, Rhys Peploe, Sauman Singh-Phulgenda, Makoto Saito, Philippe J Guérin, Prabin Dahal

**Affiliations:** Infectious Diseases Data Observatory, University of Oxford, Oxford, United Kingdom; Centre for Tropical Medicine and Global Health, Nuffield Department of Medicine, University of Oxford, Oxford, United Kingdom; Department of Nursing, College of Health Science and Medicine, Dilla University, Dilla, Ethiopia; Instituto René Rachou, Fiocruz Minas, Belo Horizonte, Minas Gerais, Brazil; Infectious Diseases Data Observatory, University of Oxford, Oxford, United Kingdom; Centre for Tropical Medicine and Global Health, Nuffield Department of Medicine, University of Oxford, Oxford, United Kingdom; Centre for Tropical Medicine and Global Health, Nuffield Department of Medicine, University of Oxford, Oxford, United Kingdom; Infectious Diseases Data Observatory, University of Oxford, Oxford, United Kingdom; Centre for Tropical Medicine and Global Health, Nuffield Department of Medicine, University of Oxford, Oxford, United Kingdom; Infectious Diseases Data Observatory, University of Oxford, Oxford, United Kingdom; Centre for Tropical Medicine and Global Health, Nuffield Department of Medicine, University of Oxford, Oxford, United Kingdom; Infectious Diseases Data Observatory, University of Oxford, Oxford, United Kingdom; Centre for Tropical Medicine and Global Health, Nuffield Department of Medicine, University of Oxford, Oxford, United Kingdom; Infectious Diseases Data Observatory, University of Oxford, Oxford, United Kingdom; Centre for Tropical Medicine and Global Health, Nuffield Department of Medicine, University of Oxford, Oxford, United Kingdom; Infectious Diseases Data Observatory, University of Oxford, Oxford, United Kingdom; Centre for Tropical Medicine and Global Health, Nuffield Department of Medicine, University of Oxford, Oxford, United Kingdom; Infectious Diseases Data Observatory, University of Oxford, Oxford, United Kingdom; Centre for Tropical Medicine and Global Health, Nuffield Department of Medicine, University of Oxford, Oxford, United Kingdom

**Keywords:** visceral leishmaniasis, SINAN, Brazil, relapse, mortality

## Abstract

**Background:**

Brazil accounts for >90% of visceral leishmaniasis (VL) cases reported in South America. VL is a notifiable disease in Brazil and patient records are captured in the Sistema de Informação de Agravos de Notificação (SINAN) database.

**Methods:**

We reviewed 55 723 patient records from the SINAN system (2007–2023) to present temporal trends in patient demographics, drug regimens administered, and treatment outcomes. Disease incidence was modeled using a negative binomial regression, and predictors of mortality were assessed using logistic regression.

**Results:**

The VL incidence declined 6% annually (incidence rate ratio, 0.94 [95% confidence interval {CI}, 0.92–0.97]), primarily during 2019–2023. The median age at presentation was 10 years (interquartile range [IQR], 2–33 years) in 2007 and 32 years (IQR, 7–48 years) in 2023. The proportion of relapse at presentation also increased over time; compared to 2007–2009, the odds of relapse during 2019–2023 were 2.10-fold higher (95% CI, 1.87–2.37) adjusted for human immunodeficiency virus status. The use of liposomal amphotericin B increased markedly from 6.1% during 2007–2009 to 38.1% during 2019–2023, while antimony use declined from 68.0% to 32.7% over the same period. Following treatment, death from VL was highest among infants (<1y) (425/4125 [10.3%]) and those >50 years of age (1471/7615 [19.3%]), compared to the other age groups (3.5%, 2.2%, and 8.0% among ages ≥1 to <5, ≥5 to <15, and ≥15 to ≤50 years, respectively).

**Conclusions:**

The shift toward older patients and increased relapse at presentation indicates a major change in disease epidemiology in Brazil. These findings highlight the need for prioritizing interventions for older and relapsing patients.

Globally, visceral leishmaniasis (VL) is endemic in 4 regions: the Indian subcontinent, Eastern Africa, the Mediterranean region, and South America [1]. In South America, the transmission of the disease is zoonotic with *Leishmania infantum* as the causative parasite species and dogs as the main reservoir [2]. In particular, >90% of the reported VL cases in the region are accounted for by Brazil.

Approximately 3500 cases are reported annually in Brazil [2, 3]; the disease disproportionately affects the Northeast regions and is strongly linked to poverty, malnutrition, environmental changes, and migration patterns [4]. Over the past 3 decades, the endemicity of disease has expanded from rural areas in the North and Northeast regions to many urban areas and newly developed cities [2, 5]. VL thus continues to be a major public health concern in the country and is listed as one of the notifiable diseases. This means that all cases coming in contact with the health system are required to be reported to the national surveillance project, the Sistema de Informação de Agravos de Notificação (SINAN) [6].

The SINAN system uses a harmonized case report form for capturing key patient details [7]. A pseudonymized version of the records captured within the system is available as an open-access resource [6]. Many studies have utilized this unique database to characterize different aspects of disease epidemiology in Brazil, such as mapping the geographical distribution of cases and mortality [3, 4, 8]. However, a comprehensive description of the disease characteristics, patient demographics, and treatment outcomes over time is currently lacking. This is particularly important as there have been several changes in the treatment strategy in the country over the past 20 years (Table 1). In this study, we describe the spatiotemporal distribution of VL cases and summarize temporal trends in patients’ characteristics, drug regimens used, and treatment outcomes using the SINAN database.

**Table 1.** Timeline of First-line Therapy for Management of Visceral Leishmaniasis in Brazil Over the Study Period

Timeline	Management
2007–2012	
First-line	PA (20 mg/kg/d, IV/IM, 20–30 d) for uncomplicated cases [26]
Alternatives	ABD (0.7–1 mg/kg/d, 15–20 d) for severe cases; L-AmB (3–5 mg/kg/d, 3–5 d) for HIV coinfection and pregnancy (limited availability) [26]
2013–2018	
First-line	PA continued; lower doses (10–15 mg/kg/d) for elderly [27]
Alternatives	Increased use of L-AmB (18–24 mg/kg total dose) for HIV patients and pregnant women [27]
2019–2025	
First-line	PA for uncomplicated cases [10]
Alternatives	L-AmB (20–40 mg/kg total dose) preferred for HIV, pregnancy, and severe cases [10]

Abbreviations: ABD, amphotericin B deoxycholate; HIV, human immunodeficiency virus; IM, intramuscular; IV, intravenous; L-AmB, liposomal amphotericin B; PA, pentavalent antimony.

## METHODS

### Study Setting and Population

Brazil is divided into 5 geographical regions: North, Northeast, Central-West, Southeast, and the South. It comprises 27 federative units, that is, 1 federal district and 26 states. The states are further divided into 5570 municipalities with a total population of approximately 212 million in 2024 [9].

### Data Source

Data from 2007 to 2023 inclusive were obtained through the SINAN portal of the Brazilian Ministry of Health, accessed via the Department of Informatics of the Unified Health System (DATASUS) [6]. Only cases marked as confirmed were included for this analysis.

### Statistical Analyses

#### Case Presentation and Clinical Characteristics

The analysis included a comprehensive set of variables encompassing patient demographics, geographical information such as federative unit, and clinical characteristics. Clinical variables covered key aspects such as reported signs and symptoms at presentation, diagnostic methods used for disease confirmation, time when treatment was initiated, drug regimen administered, and treatment outcomes. Temporal trends in VL incidence, age and sex distribution, relapse frequency, VL–human immunodeficiency virus (HIV) coinfection, and treatment regimen(s) used were summarized.

#### VL Incidence Rate and Spatiotemporal Visualization

Spatial distribution of VL cases was visualized as a choropleth using annual incidence rate at each federative unit. The annual incidence rate was computed as the number of confirmed cases divided by the population at risk, multiplied by 100 000. The choropleth maps were presented across 4 time periods (2007–2009, 2010–2014, 2015–2018, and 2019–2023) to reflect distinct epidemiological and programmatic phases. For each federative unit and time period, the mean annual incidence was computed to account for year-to-year variation. Transmission intensity was classified based on the Ministry of Health criteria: no transmission (mean = 0 cases), sporadic (mean >0 to <2.4 cases), moderate (mean ≥2.4 to <4.4 cases), and intense (mean ≥4.4 cases) [10]. Population demographic data was obtained from the Brazilian Institute of Geography and Statistics [9]. The shape file for administrative boundaries was obtained using *geobr* R package [11].

#### Regression Modeling

Disease incidence over time was modeled using a negative binomial regression; incidence rate ratios (IRRs) and corresponding 95% confidence intervals (CIs) for every calendar year increase were reported. Linear mixed-effects regression with random intercepts across federative units was applied to evaluate the association between time period and patient age at enrollment. To assess if the signs and symptoms differed by age, the association between patient's age (after scaling) and clinical signs and symptoms at presentation was investigated using a mixed-effects logistic regression with federative unit included as a random intercept to account for clustering. For predictors of mortality, univariable and multivariable logistic regression models assessed age, sex, HIV status, treatment regimen, time period, and the case type at presentation (relapse, new, transferred, or other). All analyses were performed using R statistical software version 4.3.2 [12].

#### Ethical Considerations

This study used open-access data available through the DATASUS platform of the Brazilian Ministry of Health. As the pseudonymized dataset contained no information that could identify individuals, such as names or addresses, and posed no risk to the study population, approval from a Research Ethics Committee was not required, in accordance with Resolution No. 466/2012 of the National Health Council, dated 12 December 2012 [13].

## RESULTS

### Disease Incidence and Spatiotemporal Trends

**Figure 1.**
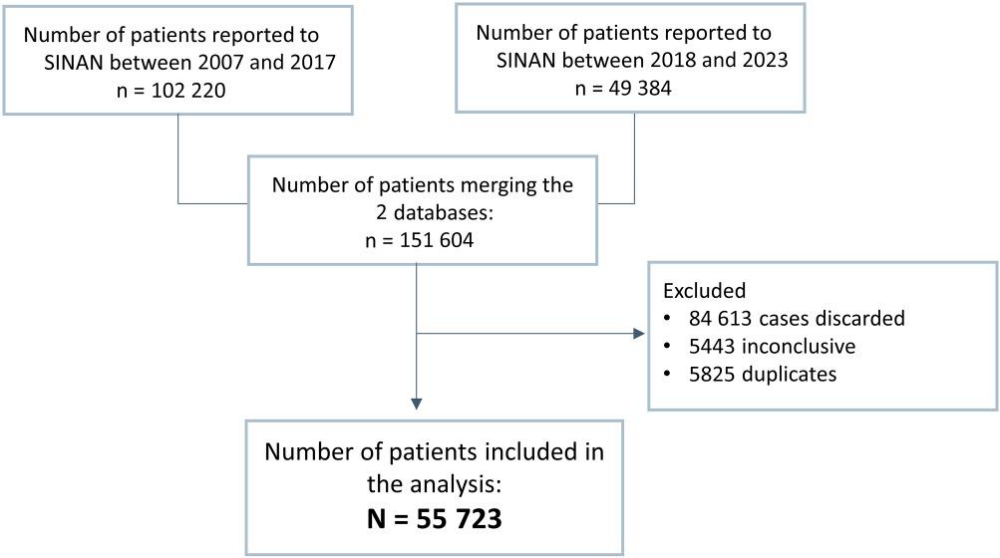
Identification of patients from the Sistema de Informação de Agravos de Notificação (SINAN) database for inclusion in the analysis. Discarded cases are those where visceral leishmaniasis (VL) was ruled out, usually due to negative laboratory results or alternative diagnoses. Inconclusive cases occur when the investigation could not confirm or exclude VL, often because of missing tests or loss to follow-up. Duplicated cases arise when the same patient is reported more than once, such as during referrals between facilities. For epidemiological analyses, only confirmed cases (regardless of availability of further diagnostic breakdown reported on exact methods used) are included, while these categories are excluded to avoid misclassification and overestimation [11].

Between 2007 and 2023, a total of 55 723 confirmed VL cases were reported (Figure 1). Disease status was confirmed using a laboratory method in 86.2% (48 038/55 723) and clinical and epidemiological method in 13.8% (7670/55 723), and this was missing in 0.02% (15/55 723). The annual incidence rate of the VL (per 100 000 inhabitants) was 0.54 (95% CI, 0.53–0.55) during 2007–2009, 0.48 (95% CI, 0.47–0.49) during 2010–2014, 0.49 (95% CI, 0.48–0.50) during 2015–2018, and 0.21 (95% CI, 0.20–0.21) during 2019–2023. Each additional calendar year was associated with an average decrease in annual case incidence by 6% (IRR, 0.94 [95% CI, 0.92–0.97]).

**Figure 2.**
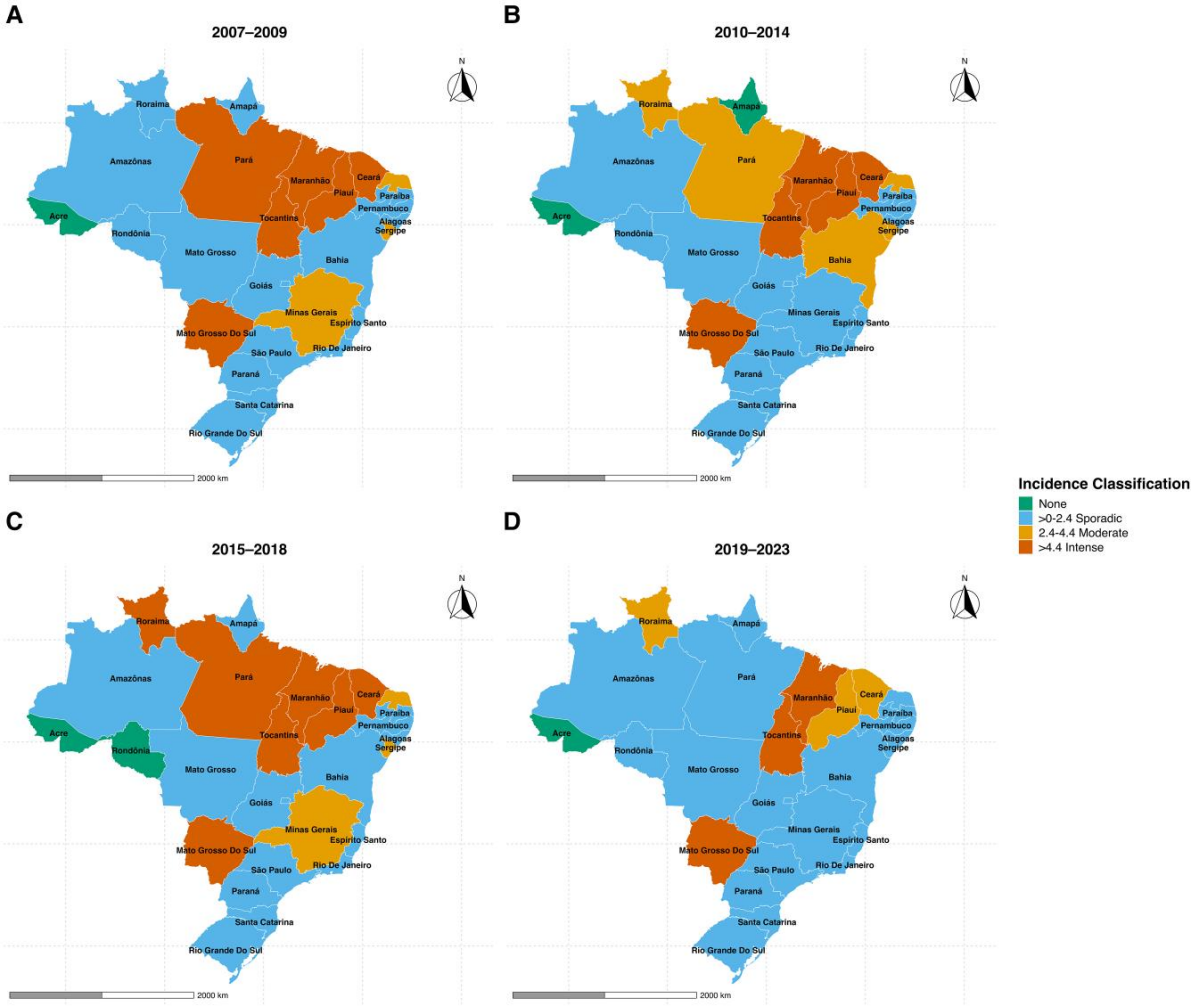
Incidence rate of visceral leishmaniasis per 100 000 population during 2007–2009 (panel *A*), 2010–2014 (panel *B*), 2015–2018 (panel *C*), and 2019–2023 (panel *D*).

Overall, 39.3% (21 884) of the total cases were reported from a few federative units, including Ceará, Minas Gerais, and Maranhão. The average annual spatiotemporal incidence rate identified Tocantins as the federative unit with consistently highest VL incidence rate, followed by Maranhão and Mato Grosso Do Sul, with an intense average annual incidence (Figure 2). This was followed by Piauí, Ceará, and Pará. A notable surge occurred between 2015 and 2018 in Pará, Bahia, and Minas Gerais, followed by a subsequent decline during 2019–2023 (Figure 2).

### Patient Characteristics

**Figure 3.**
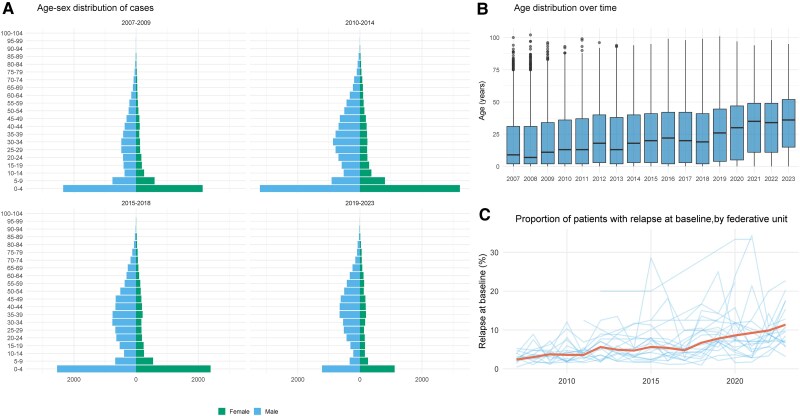
Age-sex distribution of patients and the incidence of relapse over calendar year (2007–2023). Panel *A*, Age-sex pyramid of the cases stratified by time period. Panel *B*, Box plot presenting the age distribution for each calendar year. Panel *C*, Percentage of patients reported as presenting with a relapse at baseline for each federative unit (blue) and the overall trend (red).

**Figure 4.**
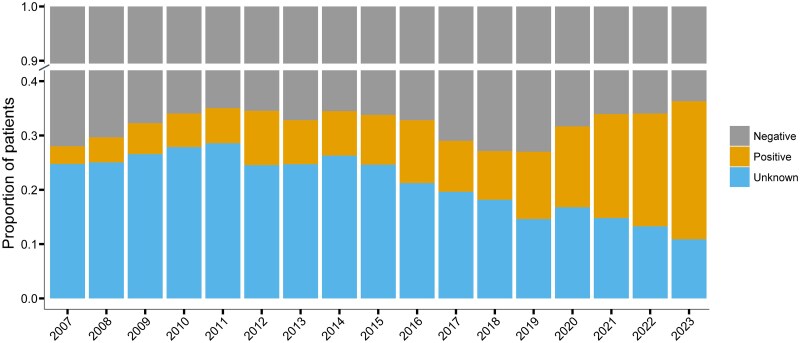
Evolution of visceral leishmaniasis/human immunodeficiency virus coinfection, by calendar year (2007–2023). The y-axis is discontinuous.

The overall median age at presentation was 19 years (interquartile range [IQR], 3–41 years) with 8.5% (4741/55 723) of the patients aged <1 year, 24.2% (13 479/55 723) ≥1 to <5 years, 13.3% (7411/55 723) ≥5 to <15 years, 38.4% (21 417/55 723) ≥15 to ≤50 years, and 15.6% (8672/55 723) >50 years (Table 2). The median age of the patients increased steadily over the study period (2007–2023): from 10 years (IQR, 2–33 years) in 2007 to 32 years (IQR, 7–48 years) in 2023 (Figure 3). In a linear regression model, compared to the reference period of 2007–2010, patients were older by 3.4 years (95% CI, 2.9–3.9 years) during 2010–2014, by 6.4 years (95% CI, 5.9–6.9 years) during 2015–2018, and by 12.5 years (95% CI, 11.9–13.0 years) during 2019–2023. The increasing trend in age over time was evident generally across all federative units (Supplementary Figure 1). A total of 36 123 patients (64.8%) were male (Table 2); this proportion remained approximately stable throughout the study period of 2007–2023 (Supplementary Figure 2). However, the sex ratio differed across age groups; the ratio was nearly balanced among infants (52.3% males) and children aged ≥1 to <5 years (50.8% males) whereas male predominance was observed with progressively increasing age (≥5 to <15 years, 55.9%; ≥15 to ≤50 years, 75.8%; and >50 years, 74.0%) (Figure 3; Table 2). The proportion of patients presenting with HIV coinfections also increased during this period (Figure 4).

**Table 2.** Baseline Patient Characteristics

Characteristic	Age <1 y (n = 4741)	Age ≥1 to <5 y (n = 13 479)	Age ≥5 to <15 y (n = 7411)	Age ≥15 to ≤50 y (n = 21 417)	Age >50 y (n = 8672)	Overall (n = 55 723)
Sex						
Female	2261 (47.7)	6635 (49.2)	3268 (44.1)	5180 (24.2)	2251 (26.0)	19 596 (35.2)
Male	2479 (52.3)	6843 (50.8)	4143 (55.9)	16 235 (75.8)	6421 (74.0)	36 123 (64.8)
Race						
Brown	3293 (69.5)	9626 (71.4)	5257 (70.9)	14 809 (69.1)	5394 (62.2)	38 380 (69.0)
White	801 (16.9)	1829 (13.6)	864 (11.7)	2712 (12.7)	1657 (19.1)	7863 (14.1)
Black	195 (4.1)	739 (5.5)	603 (8.1)	2238 (10.4)	763 (8.8)	4540 (8.1)
Indigenous	100 (2.1)	235 (1.7)	76 (1.0)	129 (0.6)	32 (0.4)	572 (1.0)
Asian Brazilian	16 (0.3)	85 (0.6)	57 (0.8)	146 (0.7)	104 (1.2)	408 (0.7)
Unknown	336 (7.1)	965 (7.2)	554 (7.5)	1383 (6.5)	722 (8.3)	3960 (7.1)
HIV status						
Negative	3511 (74.1)	9712 (72.1)	5261 (71.0)	13 028 (60.8)	5867 (67.7)	37 380 (67.1)
Positive	59 (1.2)	129 (1.0)	106 (1.4)	4073 (19.0)	910 (10.5)	5278 (9.5)
Unknown	1171 (24.7)	3638 (27.0)	2044 (27.6)	4316 (20.2)	1895 (21.8)	13 065 (23.4)
Autochthonous cases						
No	226 (4.8)	754 (5.6)	454 (6.1)	1727 (8.1)	576 (7.2)	3738 (6.7)
Yes	4149 (87.5)	11 609 (86.1)	6285 (84.8)	16 954 (79.2)	6952 (78.5)	45 950 (82.5)
Unknown	366 (7.7)	1116 (8.3)	672 (9.1)	2737 (12.7)	1144 (14.3)	6035 (10.8)
Type of case at presentation						
New	4306 (90.8)	12 186 (90.4)	6835 (92.2)	19 000 (88.7)	7769 (89.5)	50 098 (90.0)
Relapse	159 (3.4)	598 (4.4)	206 (2.8)	1471 (6.9)	537 (6.2)	2972 (5.3)
Transferred	100 (2.1)	253 (1.9)	125 (1.7)	276 (1.3)	92 (1.1)	846 (1.5)
Other	176 (3.7)	442 (3.3)	245 (3.3)	670 (3.1)	274 (3.2)	1807 (3.2)

Data are presented as No. (column %). All percentages are calculated using the total of non-missing records as the denominator.

Abbreviation: No, number of patients; HIV, human immunodeficiency virus.

### Signs and Symptoms at Presentation by Age

Signs and symptoms at presentation included fever (89.6%), weakness (77.2%), splenomegaly (73.2%), weight loss (68.8%), hepatomegaly (62.7%), and pallor (68.9%); all other reported signs and symptoms occurred in <50% of the patients (Table 3). There were some notable age-specific trends; each standard deviation increase in patient's age was associated with lower odds of fever (OR, 0.40 [95% CI, 0.38–0.41]; *P* < .001), cough and/or diarrhea (OR, 0.89 [95% CI, 0.87–0.92]; *P* < .001), pallor (OR, 0.74 [95% CI, 0.72–0.76]; *P* < .001), spleen enlargement (OR, 0.54 [95% CI, 0.52–0.55]; *P* < .001), and enlarged liver (OR, 0.64 [95% CI, 0.62–0.66]; *P* < .001). Similarly, odds of presenting with the following signs and symptoms increased with age: weakness (OR, 1.95 [95% CI, 1.88–2.02]; *P* < .001), weight loss (OR, 1.75 [95% CI, 1.69–1.80]; *P* < .001), bleeding (OR, 1.43 [95% CI, 1.37–1.48]; *P* < .001), jaundice (OR, 1.17 [95% CI, 1.14–1.21]; *P* < .001), increased infection (OR, 1.05 [95% CI, 1.02–1.08]; *P* = .003), and edema (OR, 1.09 [95% CI, 1.06–1.12]; *P* < .001).

**Table 3.** Signs and Symptoms at Presentation

Characteristic	Age <1 y (n = 4741)	Age ≥1 to <5 y (n = 13 479)	Age ≥5 to <15 y (n = 7411)	Age ≥15 to ≤50 y (n = 21 417)	Age >50 y (n = 8672)	Missing Age (n = 3)	Overall (n = 55 723)	Odds Ratio (95% CI)^a^
Fever	4486 (94.6)	12 851 (95.3)	6888 (92.9)	18 706 (87.3)	6993 (80.6)	2 (66.7)	49 926 (89.6)	0.40 (.38–.41)
Spleen enlargement	3823 (80.6)	10 919 (81.0)	5795 (78.2)	14 984 (70.0)	5276 (60.8)	2 (66.7)	40 799 (73.2)	0.54 (.52–.55)
Pallor	3539 (74.6)	9990 (74.1)	5384 (72.6)	14 140 (66.0)	5315 (61.3)	1 (33.3)	38 369 (68.9)	0.74 (.72–.76)
Liver enlargement	3284 (69.3)	9437 (70.0)	4877 (65.8)	12 720 (59.4)	4608 (53.1)	2 (66.7)	34 928 (62.7)	0.64 (.62–.66)
Weakness	2985 (63.0)	9356 (69.4)	5572 (75.2)	17 859 (83.4)	7241 (83.5)	1 (33.3)	43 014 (77.2)	1.95 (1.88–2.02)
Weight loss	2402 (50.7)	7907 (58.7)	5073 (68.4)	16 568 (77.4)	6377 (73.5)	1 (33.3)	38 328 (68.8)	1.75 (1.69–1.80)
Cough and/or diarrhea	2455 (51.8)	5987 (44.4)	2649 (35.7)	8946 (41.8)	3218 (37.1)	1 (33.3)	23 256 (41.7)	0.89 (.87–.92)
Edema	1275 (26.9)	3543 (26.3)	1518 (20.5)	4854 (22.7)	2312 (26.7)	1 (33.3)	13 503 (24.2)	1.09 (1.06–1.12)
Increased infection	1250 (26.4)	3082 (22.9)	1464 (19.8)	5038 (23.5)	2210 (25.5)	…	13 044 (23.4)	1.05 (1.02–1.08)
Jaundice	999 (21.1)	2680 (19.9)	1577 (21.3)	5491 (25.6)	1971 (22.7)	…	12 698 (22.8)	1.17 (1.14–1.21)
Bleeding	394 (8.3)	811 (6.0)	593 (8.0)	2782 (13.0)	1142 (13.2)	…	5722 (10.3)	1.43 (1.37–1.48)
Other symptoms	1011 (21.3)	2640 (19.6)	1574 (21.2)	5273 (24.6)	2374 (27.4)	1 (33.3)	12 873 (23.1)	1.16 (1.12–1.90)

Data are presented as No. (column %) unless otherwise indicated. Results are generated from a univariable logistic regression model with federative unit as a random intercept and patient’s age as the only fixed-effect covariate. Age was scaled to achieve model convergence.

Abbreviation: No., number of patients; CI, confidence interval.

^a^Odds ratio for every standard deviation increase in patient's age.

### Drug Regimen Usage Over Time

Across the study time period, the reporting forms listed pentavalent antimony (PA) as the most frequently prescribed treatment, accounting for 52.7% (29 341/55 723) of the patients. This was followed by liposomal amphotericin B (L-AmB), which was administered in 20.8% (11 613/55 723), amphotericin B deoxycholate (ABD) in 10.9% (6072/55 723), and pentamidine in 0.2% (125/55 723). Treatment was recorded as administered in the form, but drug information was missing in 6.7% (3738/55 723), and treatment status was missing for 8.7% (4834/55 723) (Table 4).

**Table 4.** Drug Regimen Administered

Characteristic	All (n = 55 723)	PA (n = 29 341)	L-AmB (n = 11 613)	ABD (n = 6072)	Pentamidine (n = 125)	Treated but Not Documented (n = 3738)	Treatment Status Missing (n = 4834)
Age group							
<1 y	4741	2082 (43.9)	1400 (29.5)	672 (14.2)	7 (0.1)	221 (4.7)	359 (7.6)
≥1 to <5 y	13 479	9172 (68.0)	1591 (11.8)	947 (7.0)	23 (0.2)	699 (5.2)	1047 (7.8)
≥5 to <15 y	7411	5239 (70.7)	619 (8.4)	447 (6.0)	21 (0.3)	436 (5.9)	649 (8.7)
≥15 to ≤50 y	21 417	10 561 (49.3)	4586 (21.4)	2831 (13.2)	56 (0.3)	1509 (7.0)	1874 (8.8)
>50 y	8672	2287 (26.4)	3416 (39.4)	1175 (13.5)	16 (0.2)	873 (10.1)	905 (10.4)
Missing age	3	0 (0)	1 (33.3)	0 (0)	2 (66.7)	0 (0)	0 (0)
Time period							
2007–2009	11 450	7782 (68.0)	695 (6.1)	1155 (10.1)	21 (0.2)	520 (4.5)	1277 (11.1)
2010–2014	18 285	10 634 (58.2)	2827 (15.5)	2383 (13.0)	42 (0.2)	1140 (6.2)	1259 (6.9)
2015–2018	15 320	7434 (48.5)	4023 (26.3)	1474 (9.6)	25 (0.2)	1183 (7.7)	1181 (7.7)
2019–2023	10 665	3491 (32.7)	4066 (38.1)	1060 (9.9)	36 (0.3)	895 (8.4)	1117 (10.6)
Type of case at presentation							
New cases	50 098	27 886 (55.7)	9979 (19.9)	5380 (10.7)	89 (0.2)	3347 (6.7)	3417 (6.8)
Relapse	2972	748 (25.2)	1426 (48.0)	455 (15.3)	29 (1.0)	146 (4.9)	168 (5.6)
Transferred cases	846	434 (51.3)	128 (15.1)	124 (14.7)	4 (0.5)	73 (8.6)	83 (9.8)
Other	1807	273 (15.1)	80 (4.4)	113 (6.3)	3 (0.2)	172 (9.5)	1166 (64.5)
HIV status							
Negative	37 380	21 216 (56.8)	7303 (19.5)	4088 (10.9)	64 (0.2)	2478 (6.6)	2231 (6.0)
Positive	5278	827 (15.7)	2884 (54.6)	972 (18.4)	33 (0.6)	281 (5.3)	281 (5.4)
Unknown	13 065	7298 (55.9)	1426 (10.9)	1012 (7.7)	28 (0.2)	979 (7.5)	2322 (17.8)

Data are presented as No. (row %).

Abbreviations: No, number of patients; ABD, amphotericin B deoxycholate; HIV, human immunodeficiency virus; L-AmB, liposomal amphotericin B; PA, pentavalent antimony.

**Figure 5.**
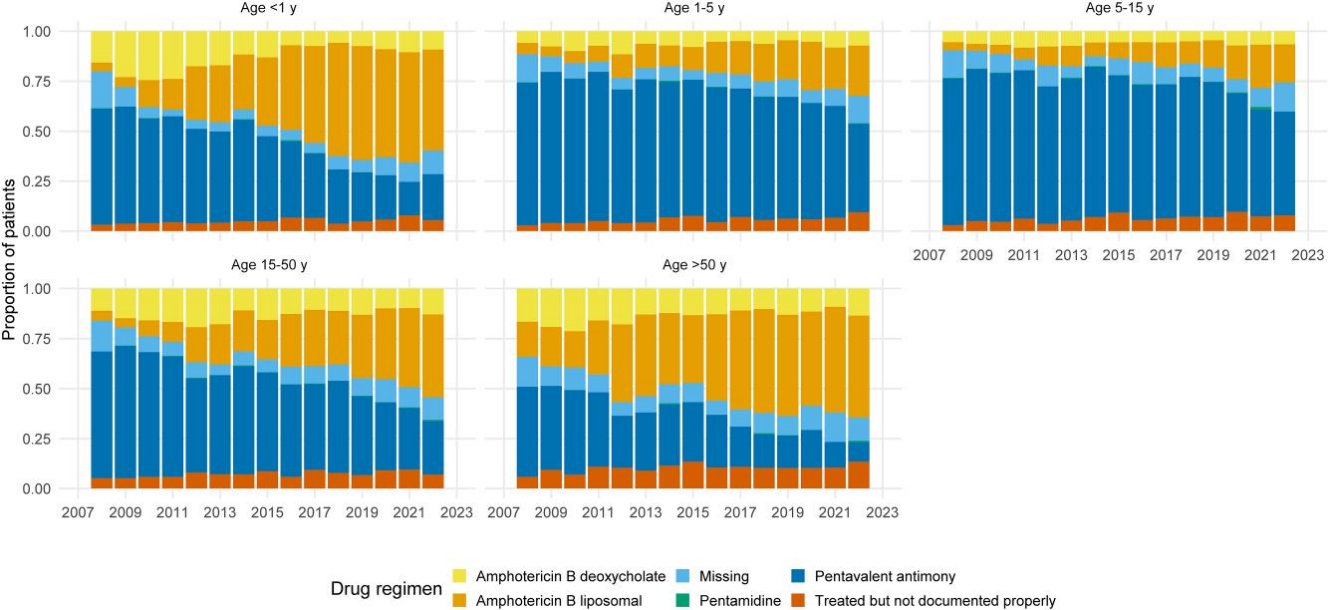
A stacked bar chart showing the proportion of patients receiving different drug regimens by age group over the study period. 1 – 5 y indicate age range ≥1 to <5 years, 5 – 15 y indicate age range ≥5 to <15 years, and 15 – 50 y indicate age range ≥15 to ≤50 years

There were some noticeable changes in drug usage over time, particularly with variation across different age groups, likely reflecting changes in drug policy (Figure 5 and Table 1); the use of L-AmB progressively increased from 6.1% during 2007–2009 to 38.1% during 2019–2023, and these figures corresponded with a decline in antimony usage from 68.0% during 2007–2009 to 32.7% during 2019–2023 (Table 4). The progressive evolution in the adoption of treatment regimens across different age groups is further presented in Figure 5. The management of patients with HIV coinfection largely relied on amphotericin-based regimens, with 54.6% receiving L-AmB (2884/5278) and 18.4% receiving ABD (972/5278) (Table 4).

### Rising Trend of Relapse (n = 2972)

At presentation, 90.0% (50 098/55 723) of the patients were reported as new cases (primary VL), 5.3% (2972/55 723) as relapses, 1.5% (846/55 723) as transferred cases from another health service, and 3.3% (1807/55 723) as “other” (Table 2); the latter 2 categories reflect cases with unavailable information. Relapse cases at presentation increased over time and were reported in 3.1% (465/15 154) during 2007–2009, 4.6% (678/14 581) during 2010–2014, 5.6% (854/15 320) during 2015–2018, and 9.1% (974/10 665) during 2019–2023 (Figure 3). In a multivariable logistic regression adjusted for HIV status, compared to the reference period of 2007–2009, the odds of presenting with a relapse increased by 1.36-fold (95% CI, 1.20–1.54) during 2010–2014, 1.55-fold (95% CI, 1.37–1.74) during 2015–2018, and 2.10-fold (95% CI, 1.87–2.37) during 2019–2023. This increasing trend was apparent across almost all the federative units (Figure 3). HIV-positive patients accounted for 22.1% (1167/2972) of all relapse cases, and there was a general increase in the proportion of relapse among HIV-coinfected patients over time (Supplementary Figure 3).

### Treatment Outcomes

Treatment outcomes were available for 48 073 of the 55 723 (86.3%) confirmed cases; outcome information was missing for 7650 (13.7%). Overall, 80.9% (38 937/48 073) of patients were cured, 8.2% (3925/48 073) died from causes considered likely related to VL, and 2.7% (1311/48 073) died from causes not classified as directly related to VL (Table 5). Treatment outcomes by type of treatment received are presented in Supplementary Figures 3–5.

**Table 5.** Treatment Outcomes

Characteristic^a^	No.	Abandonment	Cured	Death From VL	Death From Other Causes	Transfer
Age group						
<1 y	4125	11 (0.3)	3293 (79.8)	425 (10.3)	53 (1.3)	343 (8.3)
≥1 to <5 y	11 567	41 (0.4)	9919 (85.8)	410 (3.5)	54 (0.5)	1143 (9.8)
≥5 to <15 y	6302	21 (0.3)	5579 (88.5)	141 (2.2)	33 (0.5)	528 (8.5)
≥15 to ≤50 y	18 462	296 (1.6)	14 957 (81.0)	1478 (8.0)	639 (3.5)	1092 (5.9)
>50 y	7615	66 (0.9)	5188 (68.1)	1471 (19.3)	532 (7.0)	358 (4.7)
Missing	2	0 (0)	1 (50.0)	0 (0)	0 (0)	1 (50.0)
Drug regimen						
Amphotericin B deoxycholate	5466	38 (0.7)	4257 (77.9)	707 (12.9)	183 (3.3)	281 (5.2)
Liposomal amphotericin B	10 493	64 (0.6)	8581 (81.8)	1159 (11.0)	381 (3.6)	308 (3.0)
Treatment status missing	3326	71 (2.1)	2164 (65.1)	430 (12.9)	195 (5.9)	466 (14.0)
Pentamidine	113	0 (0)	101 (89.4)	6 (5.3)	1 (0.9)	5 (4.4)
Pentavalent antimony	25 487	167 (0.7)	21 932 (86.1)	1019 (4.0)	268 (1.1)	2101 (8.1)
Treated but drug not documented	3188	95 (3.0)	1902 (59.7)	604 (18.9)	283 (8.9)	304 (9.5)
Time period						
2007–2009	13 147	67 (0.7)	8111 (82.4)	649 (6.7)	179 (1.9)	840 (8.3)
2010–2014	12 691	134 (0.8)	13 043 (81.3)	1196 (7.6)	385 (2.4)	1234 (7.9)
2015–2018	13 253	118 (0.9)	10 692 (80.7)	1188 (9.0)	405 (3.1)	850 (6.3)
2019–2023	8980	116 (1.3)	7090 (79.0)	892 (9.9)	342 (3.8)	540 (6.0)
Missing	2	0 (0)	1 (50.0)	0 (0)	0 (0)	1 (50.0)
HIV status						
HIV negative	32 620	255 (0.8)	26 912 (82.5)	2570 (7.9)	625 (1.9)	2240 (6.9)
HIV positive	4724	97 (2.1)	3531 (74.7)	452 (9.6)	412 (8.7)	232 (4.9)
HIV status unknown	10 747	83 (0.8)	8494 (79.0)	903 (8.4)	274 (2.5)	993 (9.3)
Type of case at baseline						
New case	53 510	372 (0.9)	35 371 (81.3)	3566 (8.2)	1153 (2.6)	3048 (7.0)
Relapse case	2585	47 (1.8)	2118 (81.9)	162 (6.3)	88 (3.4)	170 (6.6)
Other	1250	10 (0.8)	944 (75.5)	127 (10.2)	54 (4.3)	115 (9.2)
Transferred case	728	6 (0.8)	504 (69.2)	70 (9.6)	16 (2.2)	132 (18.3)
Overall	48 073	435 (0.1)	38 937 (80.9)	3925 (8.2)	1311 (2.7)	3465 (7.1)

Data are presented as No. (row %).

Abbreviations: No.= Number of patients; HIV, human immunodeficiency virus; VL, visceral leishmaniasis.

^a^Percentages were calculated using only cases with known treatment outcomes as the denominator.

Of the total cases, 52.7% (29 341/55 723) of patients were treated with PA. Among those receiving PA, treatment failure was observed in 8.4% (860/10 134) during 2007–2009, 10.5% (870/8282) during 2010–2014, 13.8% (1026/7434) during 2015–2019, and 16.4% (573/3491) during 2019–2023 across successive year ranges, indicating a steadily increasing trend in PA treatment failure over time (Supplementary Figure 3).

### Mortality

Death from VL was 10.3% among infants (425/4125), 3.5% among patients aged ≥1 to <5 years (410/11 567), 2.2% among those aged ≥5 to <15 years (141/6302), 8.0% among those aged ≥15 to ≤50 years (1478/18 462), and 19.3% for those aged >50 years (1471/7615). All-cause mortality was 11.5% among infants (478/4125), 4.0% among those aged ≥1 to <5 years (464/11 567), 2.8% among those aged ≥5 to <15 years (174/6302), 11.5% among those aged ≥15 to ≤50 years (2117/18 462), and 26.3% for those aged >50 years (2003/7615).

All-cause mortality generally increased over time in univariable analysis (Supplementary Figure 6), but the time trend was reversed when adjusted for other key covariates in a multivariable analysis, with 16% lower odds (adjusted odds ratio [AOR], 0.84 [95% CI, .76–.94]) during 2019–2023 compared with the reference period of 2007–2009 (Table 6). Children aged <1 year and patients aged >50 years were at 4.2-fold (95% CI, 3.5–5.1) and 9.8-fold (95% CI, 8.3–11.5) increased odds of all-cause mortality, compared to the age group ≥5 to <15 years. The AOR was also higher among patients with HIV coinfection (AOR, 1.56 [95% CI, 1.42–1.71]) compared to those without HIV and among patients who received ABD (AOR, 2.78 [95% CI, 2.52–3.06]) and L-AmB (AOR, 2.14 [95% CI, 1.95–2.35]) compared to those who received antimony, and was lower among patients who presented with a relapse at baseline compared to the newly diagnosed cases (AOR, 0.59 [95% CI, .51–.68]) (Table 6).

**Table 6.** Univariable and Multivariable Logistic Regression for All-Cause Mortality

Characteristic	No. of Deaths	Univariable OR (95% CI)	Multivariable OR (95% CI)^a^
Time period			
2007–2009	1138	Reference	Reference
2010–2014	1271	1.21 (1.11–1.32)	0.95 (.86–1.04)
2015–2018	1593	1.49 (1.36–1.63)	0.97 (.88–1.07)
2019–2023	1234	1.65 (1.51–1.82)	0.84 (.76–0.94)
Age group			
<1 y	478	4.78 (4.00–5.71)	4.22 (3.51–5.08)
≥1 to <5 y	464	1.49 (1.25–1.78)	1.54 (1.28–1.84)
≥5 to <15 y	174	Reference	Reference
≥15 to ≤50 y	2117	4.57 (3.9–5.35)	3.89 (3.30–4.58)
>50 y	2003	12.45 (10.62–14.6)	9.77 (8.28–11.54)
Drug regimen			
Pentavalent antimony	1287	Reference	Reference
Amphotericin B deoxycholate	890	3.72 (3.39–4.08)	2.78 (2.52–3.06)
Liposomal amphotericin B	1540	3.34 (3.08–3.62)	2.14 (1.95–2.35)
Drug information missing	625	3.27 (2.95–3.62)	2.78 (2.48–3.10)
Pentamidine	7	1.26 (.60–2.63)	1.12 (.51–2.50)
Treated but not documented properly	887	6.84 (6.22–7.52)	5.42 (4.90–6.00)
HIV status			
HIV negative	3195	Reference	Reference
HIV positive	864	2.03 (1.87–2.20)	1.56 (1.42–1.71)
HIV status unknown	1177	1.08 (1.00–1.16)	1.09 (1.01–1.17)
Type of case at baseline			
New cases	4719	Reference	Reference
Relapse cases	250	0.84 (.73–.95)	0.59 (.51–.68)
Other	181	1.08 (.92–1.26)	0.75 (.61–.93)
Transferred cases	86	1.10 (.88–1.38)	1.12 (.88–1.42)

Abbreviations: CI, confidence interval; HIV, human immunodeficiency virus; OR, odds ratio.

^a^n = 54 929 observations in the multivariable logistic regression, which included all the variables assessed in univariable analysis and no variable selection was performed. The model included federative unit as a random intercept and the intraclass correlation coefficient for random intercept was 0.009.

## DISCUSSION

Between 2007 and 2023, Brazil experienced an average annual decline in VL incidence of 6.0%; this decline occurred primarily over the latter 5 years (2019–2023) (Supplementary Figure 7). This overall decline likely reflects the combined effect of insecticide spraying for vector control, canine reservoir control, and improvements in housing and sanitation, and also likely due to the expanded use of L-AmB [14–16]. Establishment of surveillance systems such as SINAN improved case tracing and management [6]. A key point to caution is that this overall national trend may obscure regional variations (Figure 2). This is particularly relevant as VL transmission is influenced by socioenvironmental factors and spatial clustering, with incidence reductions varying by region due to heterogeneous intervention coverage and evolving ecological risks like urbanization, as observed in Tocantins and Mato Grosso do Sul [17–19].

Our analysis also identified a major shift in disease burden in the country from pediatric population toward adults as the median age of VL patients increased from 10 years in 2007 to 32 years in 2023 (Figure 3). The shift is likely due to periurban exposure and the urbanization of transmission [20]. In a sylvatic model, exposure typically occurs predominantly in childhood, and adults are relatively protected owing to prior subclinical infections and acquired immunity [21]. With the emergence of new foci of transmission in urban peripheries, infections now can occur across all age groups, including immunologically naive adults, thereby increasing the proportion of adult cases [5]. In line with previous research, our analysis also found sex disparities in VL incidence, with males comprising 64.8% of overall cases and the male predominance becoming more pronounced with increasing age (Figure 3). This likely reflects gendered occupational exposures, such as migration to high-VL-endemic areas for agricultural work [22, 23].

An increase in relapse was observed over time as the proportion of patients presenting with a relapse at baseline tripled from 3.1% to 9.1% between 2007–2009 and 2019–2023 (Figure 3). While HIV coinfection likely amplifies this trend, we found that this increase persisted even accounting for HIV status, suggesting that factors beyond immunosuppression may be contributing to relapse risk. The proportion of patients presenting with VL–HIV coinfections was also found to increase over time (Figure 4). This overall increase in relapse along with increasing incidence of VL–HIV cases underscores a growing public health concern in Brazil, a pattern that is also observed in other key endemic regions of East Africa and South Asia [24, 25]. Taken together, these findings highlight that both host (eg, HIV coinfection, immunity) and treatment factors remain important drivers of relapse, reinforcing the need for targeted interventions toward these susceptible populations.

In parallel with these shifts in patient and disease characteristics, our analysis also characterized a clear shift in treatment practices over time. A progressively increased adoption of L-AmB was observed, particularly in infants and those >15 years of age (Figure 5). This reflects the changes in national guidelines that gradually expanded the indications for L-AmB, including not only for the patients with restrictions on antimonial use, but also cases meeting severity criteria and subgroups with a recognized poorer prognosis, such as the extremes of age and immunocompromised patients, marking a positive trend toward safer and more effective therapies (Table 1) [10]. However, PA still remained the predominant regimen in Brazil, especially in those aged ≥5 to <15 years. The sustained use of antimony regimens in this patient group, despite their well-established toxicity, may warrant further review [18].

Following treatment, mortality attributed to VL was reported in approximately 8% of the patients. As expected, extremes of age emerged as a risk factor for mortality with infants (<1 year) and older adults (>50 years) identified as high-risk groups, a finding also noted in the Brazilian national guidelines and elsewhere in the literature [4, 26–29]. However, there were 2 paradoxical findings: (1) a lower mortality was observed among patients presenting with a relapse compared to those newly diagnosed VL cases, and (2) a higher mortality was observed following treatment with a L-AmB regimen compared to antimony (Table 6). The former is potentially explained by a survivorship bias, as patients who survive initial VL episodes are most likely to receive more attentive care during management of relapse, which may have resulted in a lower fatality. Disease immunology may provide additional perspective: In patients presenting with a relapse, residual parasitemia may induce a form of immune tolerance, allowing a prolonged, low-grade infection with reduced inflammatory-mediated tissue damage [26]. The second paradoxical finding of a higher case fatality rate among those treated with nonantimony regimens such as L-AmB is likely due to confounding by indication. PA is typically used for uncomplicated VL, whereas L-AmB regimens are reserved for severe and high-risk cases (eg, HIV coinfection, those presenting with a renal dysfunction, or severe symptoms like jaundice), all of which carry a higher mortality risk [4].

Our study has some limitations. First, as an observational analysis, it is subject to reporting bias and incompleteness of routine surveillance data. The reliability of some variables is of particular concern. For example, the case definition of “relapse” may have been inconsistently applied across different cases. The “treatment” variable is also likely to capture only the initial regimen and not subsequent rescue therapy. In addition, the limited number of available covariates restricted our ability to adjust for important predictors of treatment outcome, such as disease severity. Although a validated severity score for VL exists in the context of Brazil [10], not all relevant data required for estimating this score are captured in the SINAN system, which precluded the possibility of adjudicating disease severity status. Further limitation was that the definitions used for “other causes of mortality” were not fully clear, and it is uncertain whether deaths in the “other” category could include treatment-related toxicity or complications arising from VL, as no formal guidance was provided. Missing data were also a concern, as treatment outcomes were not recorded for a proportion of patients; exploring the underlying causes of such missingness remains challenging. Finally, the analysis was restricted to confirmed cases as indicated in the SINAN database and, there were some cases with diagnostic details missing despite reported as confirmed cases.

Despite these limitations, our study has important strengths. The SINAN database represents the largest single source of open-access VL patient and treatment data globally, providing a unique opportunity to examine epidemiological trends over time. The national coverage not only enhanced the generalizability of our findings within the South America, but also allowed investigating outcomes for some key patient subgroups, such as infants, which would be difficult to assess in smaller or local studies. An additional strength is that our study also highlights the value of open national surveillance data for secondary analysis, which allowed identification of epidemiological trends directly relevant to disease control and patient care. Brazil should be praised for the initiatives taken to harmonize the data collection through a centralized electronic data capture system and for its active open-science policy. This model can potentially serve as a blueprint for the disease control programs not only for other VL-endemic regions but also for disease control programs beyond VL. For funders, this also underscores the benefit of supporting data reuse and maximizing returns on existing investments by generating evidence for the common good.

## CONCLUSIONS

The observed increase in patients presenting with a VL relapse over time despite an overall decline in VL incidence, together with the rising median age of affected patients, suggests a shift in the epidemiology of the disease in Brazil. This evolving pattern is further characterized by a growing burden of VL–HIV coinfection and persistent use of antimony regimen. Such trends underscore the need for more targeted and adaptive control strategies, including integrated VL–HIV management, expanded access to safer treatments such as L-AmB, and strengthened posttreatment monitoring for relapse.

## Supplementary Material

ofag035_Supplementary_Data

## Supplementary Data

Supplementary materials are available at *Open Forum Infectious Diseases* online. Consisting of data provided by the authors to benefit the reader, the posted materials are not copyedited and are the sole responsibility of the authors, so questions or comments should be addressed to the corresponding author.
