# Supplementary material for: Epidemiological Shifts in Visceral Leishmaniasis Incidence, Relapse, and Mortality in Brazil, 2007–2023: Analysis Using the National Notifiable Diseases Information System
Source: Open Forum Infect Dis. 2026 Feb 17;13(2):ofag035. doi: 10.1093/ofid/ofag035 (PMC12910620; doi:10.1093/ofid/ofag035)
Supplement: ofag035_Supplementary_Data [file ofag035_supplementary_data.docx]

# Supplemental file:

Epidemiological shifts in visceral leishmaniasis incidence, relapse, and mortality in Brazil, 2007–2023: Analysis using the national notifiable diseases information system

Dawit Getachew Assefa^1,2,3^, Glaucia Cota^4^, James P. Wilson^1,2^, Jessica Andretta Mendes^2^,

Caitlin Naylor^1,2^, Rhys Peploe^1,2^, Sauman Singh-Phulgenda^1,2^, Makoto Saito^1,2^, Philippe J

Guerin^1,2^, Prabin Dahal^1,2*^

^1^Infectious Diseases Data Observatory (IDDO), Oxford, UK

^2^Centre for Tropical Medicine and Global Health, Nuffield Department of Medicine, University of Oxford, Oxford, UK

^3^Department of Nursing, College of Health Science and Medicine, Dilla University, Dilla, Ethiopia

^4^Instituto René Rachou, Fiocruz Minas, Belo Horizonte, Minas Gerais, Brazil

*[prabin.dahal@ndm.ox.ac.uk](mailto:prabin.dahal@ndm.ox.ac.uk)

Infectious Diseases Data Observatory (IDDO), University of Oxford, Oxford, UK

# List of supplemental figures

# Supplemental figure 1: Median age by federative units over calendar year in epidemiological shifts in visceral leishmaniasis incidence, relapse, and mortality in Brazil, 2007-2023.

# Supplemental figure 2: Age-sex distribution of cases in epidemiological shifts in visceral leishmaniasis incidence, relapse, and mortality in Brazil, 2007–2023.

# Supplemental figure 3: Trends over time in VL-HIV cases and failure against antimony drugs, in visceral leishmaniasis incidence, relapse, and mortality in Brazil, 2007–2023.

# Supplemental figure 4: Reported treatment outcomes (proportions) by drug regimens over calendar year, in visceral leishmaniasis incidence, relapse, and mortality in Brazil, 2007–2023.

# Supplemental figure 5: Reported treatment outcomes (counts) by drug regimens over

# calendar year stratified by age-groups, in visceral leishmaniasis incidence, relapse, and

# mortality in Brazil, 2007–2023. Panel (A) presents data for children aged <5 years and

# Panel (B) presents data for patients aged ≥5 years.

# Supplemental figure 6: Case fatality rate over calendar year, in visceral leishmaniasis

# incidence, relapse, and mortality in Brazil, 2007–2023.

# Supplemental figure 7: The number of confirmed cases captured in the national surveillance

# database (SINAN) over calendar year, in visceral leishmaniasis incidence, relapse, and

# mortality in Brazil, 2007–2023.


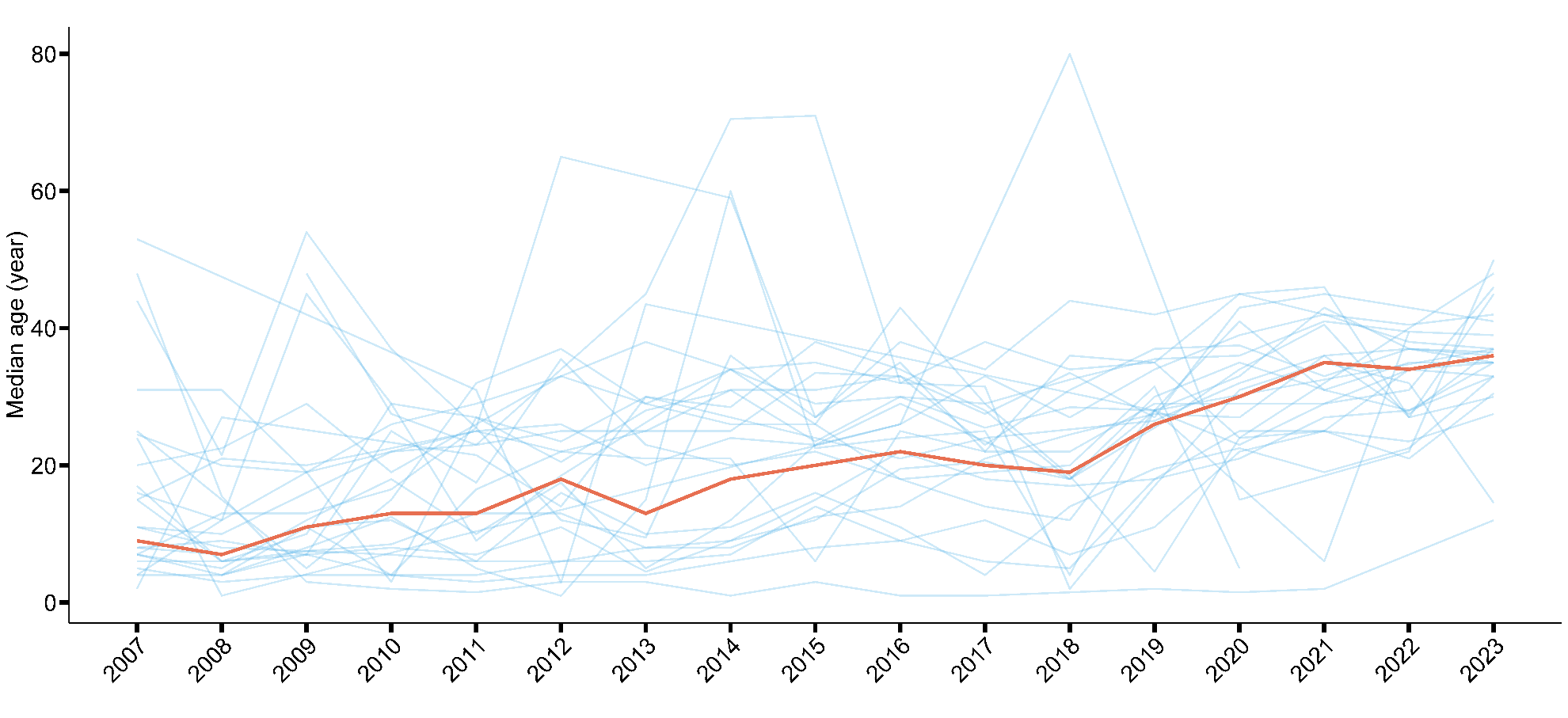


# Supplemental figure 1: Median age by federative units over calendar year in epidemiological shifts in visceral leishmaniasis incidence, relapse, and mortality in Brazil, 2007-2023.

# Legend: Each blue line represents a federative unit. The solid red line is the overall median age by year.


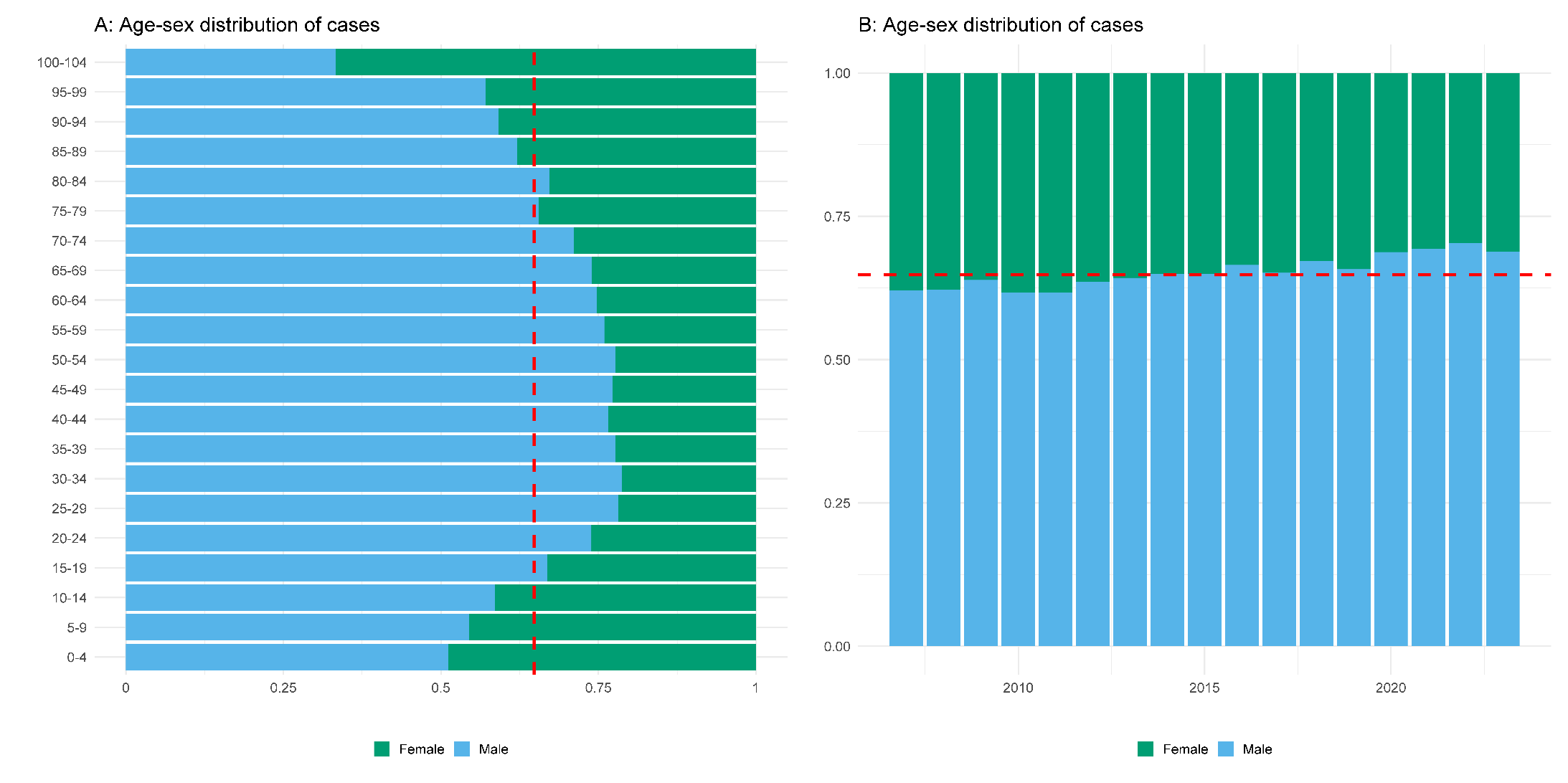


# Supplemental figure 2: Age-sex distribution of cases in epidemiological shifts in visceral leishmaniasis incidence, relapse, and mortality in Brazil, 2007–2023.

# Legend: Panel (A) presents the age-sex distribution of cases for the entire study period 2007–2023. Panel (B) presents sex-distribution by each calendar year of case notification. Red line represents the overall proportion of male (64.8%) for the entire study period, 2007–2023.


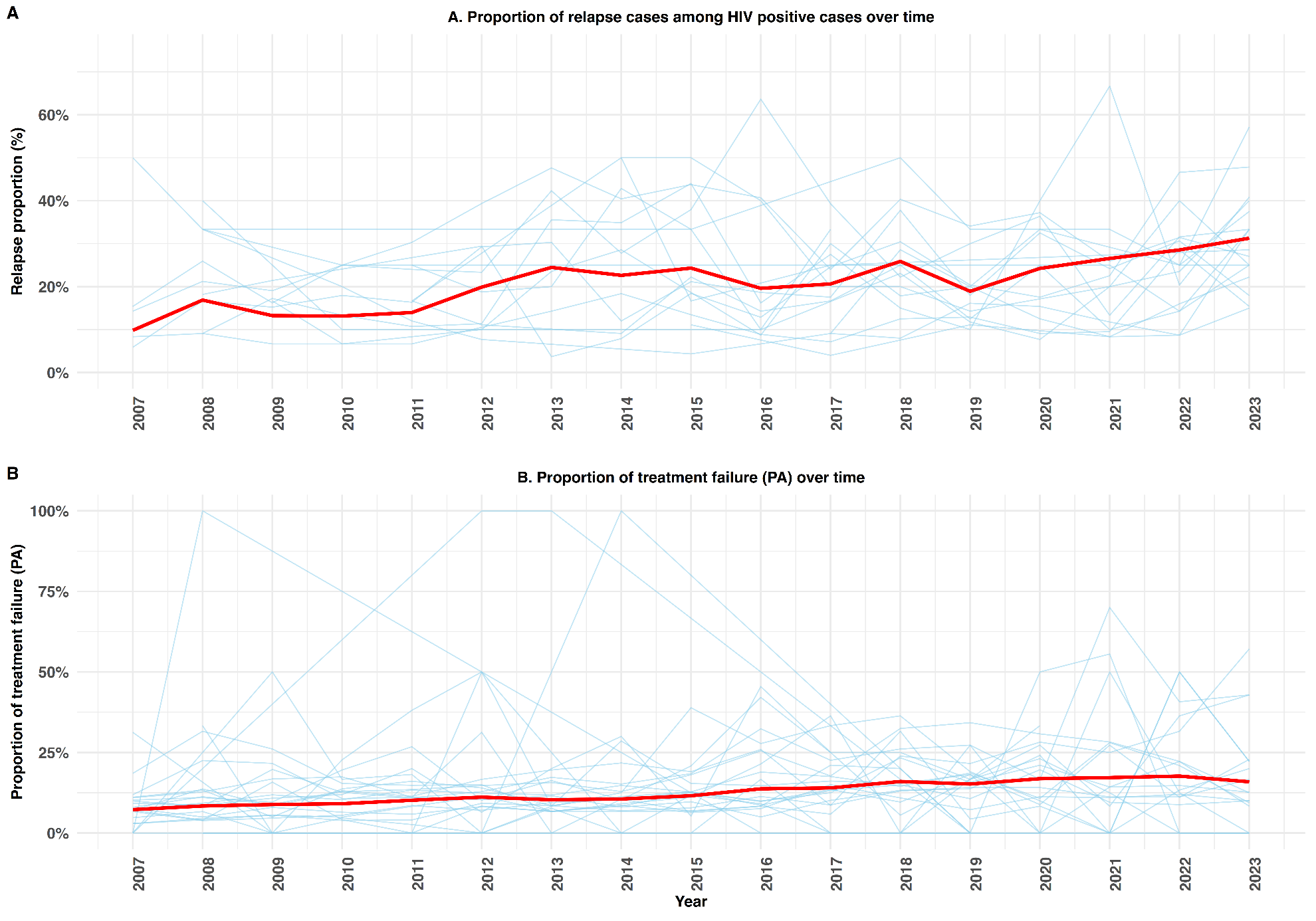


# Supplemental figure 3: Trends over time in VL-HIV cases and failure against antimony drugs, in visceral leishmaniasis incidence, relapse, and mortality in Brazil, 2007–2023.

# Legend: Panel (A) presents the distribution of relapse among HIV positive cases over calendar year. Panel (B) depicts the proportion of treatment failure to pentavalent antimony regimen over time. The solid red line is the overall percentage for each panel. The blue lines present data for each federative unit.


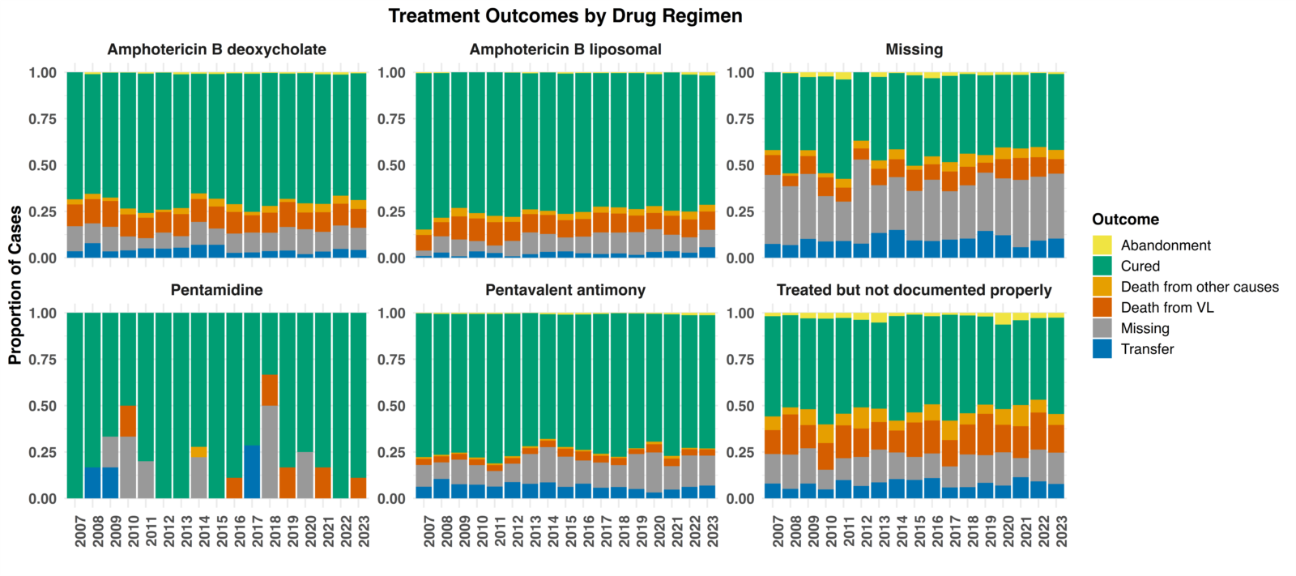


# Supplemental figure 4: Reported treatment outcomes (proportions) by drug regimens over calendar year, in visceral leishmaniasis incidence, relapse, and mortality in Brazil, 2007–2023.


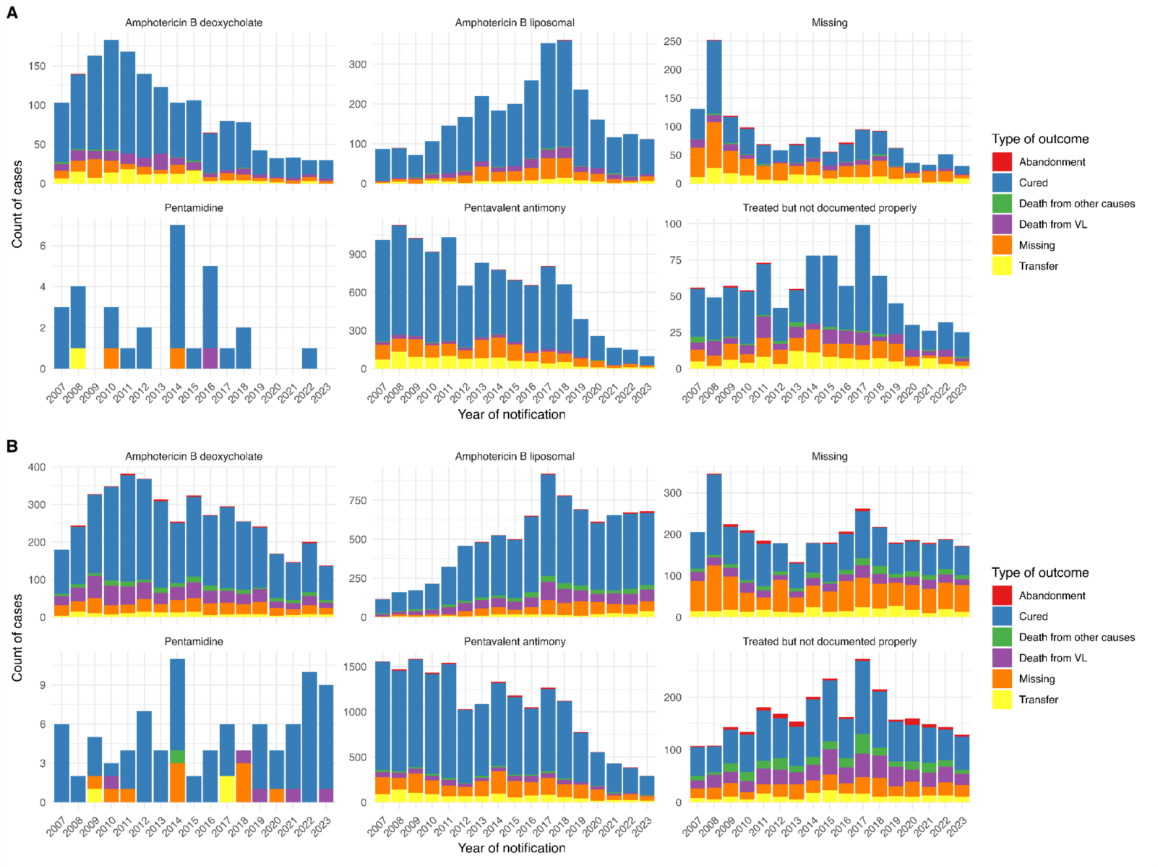


Supplemental figure 5: Reported treatment outcomes (counts) by drug regimens over calendar year stratified by age-groups, in

visceral leishmaniasis incidence, relapse, and mortality in Brazil, 2007–2023. Panel (A) presents data for children aged <5 years and

Panel (B) presents data for patients aged ≥5 years.


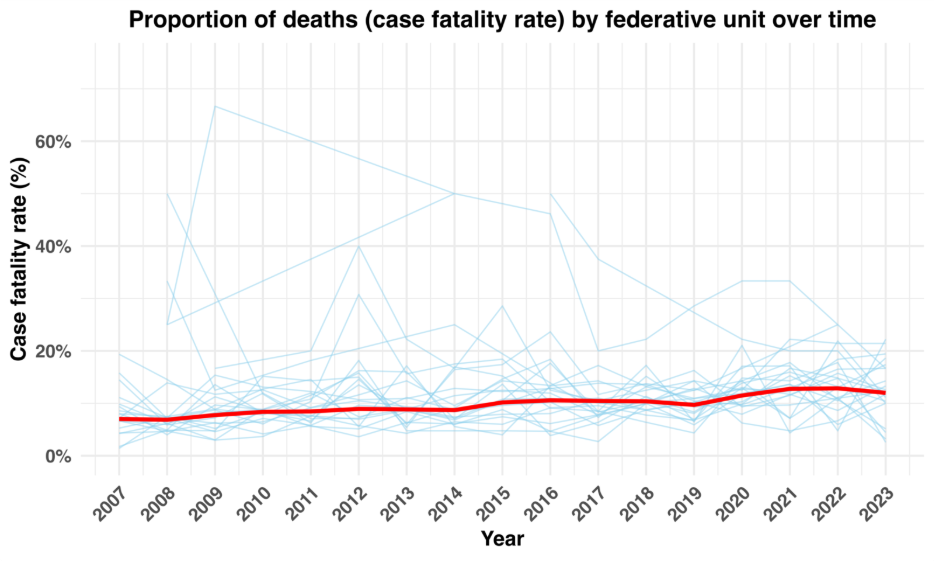


Supplemental figure 6: Case fatality rate over calendar year, in visceral leishmaniasis incidence, relapse, and mortality in Brazil,

2007–2023.

Legend: The solid red line is the overall percentage for each year.


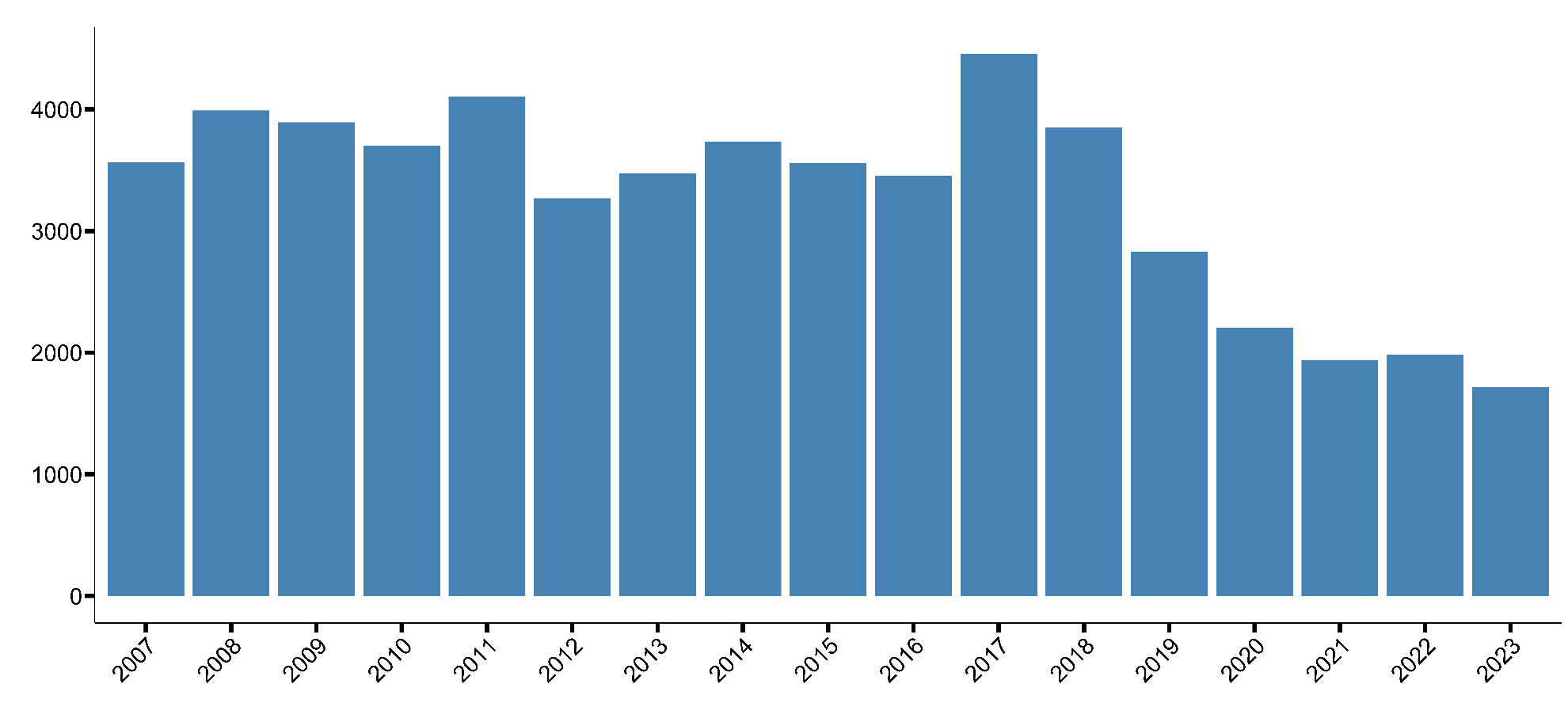


Supplemental figure 7: The number of confirmed cases captured in the national surveillance database (SINAN) over calendar year,

in visceral leishmaniasis incidence, relapse, and mortality in Brazil, 2007–2023.

Legend: Y-axis presents the number of confirmed cases and X-axis presents the calendar year of notification.
